# Application of DBN and GWO-SVM in analog circuit fault diagnosis

**DOI:** 10.1038/s41598-021-86916-6

**Published:** 2021-04-12

**Authors:** Xiyuan Su, Changqing Cao, Xiaodong Zeng, Zhejun Feng, Jingshi Shen, Xu Yan, Zengyan Wu

**Affiliations:** 1 School of Physics and Optoelectronic Engineering, Xidian University, No. 2 Taibai South Road, Xi’an, 710071 China; 2 Institute of Space Electronic Technology, Hangtian Road, Yantai, 264670 China

**Keywords:** Computer science, Electrical and electronic engineering

## Abstract

For large-scale integrated electronic equipment, the complex operating mechanisms make fault detection very difficult. Therefore, it is important to accurately identify analog circuit faults in a timely manner. To overcome this problem, this paper proposes a novel fault diagnosis method based on the deep belief network (DBN) and restricted Boltzmann machine (RBM) optimized by the gray wolf optimization (GWO) algorithm. First, DBN is used to extract the deep features of the analog circuit output signal. Then, GWO is used to optimize the penalty factor c and kernel parameter g of support vector machine (SVM). Finally, GWO-SVM is used to diagnose the signal features extracted by the DBN. Fault diagnosis simulation was conducted for the Sallen–Key band-pass filter and a four-opamp biquad highpass filter. The experimental results show that compared with the existing algorithms, the algorithm proposed in this paper improves the accuracy of Sallen–Key bandpass filter circuit to 100% and shortens the fault diagnosis time by about 90%; for four-opamp biquad highpass filter, the accuracy rate has increased to 99.68%, and the fault diagnosis time has been shortened by approximately 75%, and reduce hundreds of iterations. Moreover, the experimental results reveal that the proposed fault diagnosis method greatly improves the accuracy of analog circuit fault diagnosis, which solves a major problem in analog circuitry and has great significance for the future development of relevant applications.

## Introduction

With the rapid development of science and technology, electronic circuits are increasingly being used in communications, industrial control, medical, household appliances, aerospace, military, and many other fields. As the complexity of these electronic circuits increases with circuits becoming more integrated, the requirements and dependency on diagnostic technology has also increased accordingly. For critical electronic equipment used in important fields, the diagnostic index requirements are stricter and more comprehensive and demanding better diagnostic accuracy.

For all electronic circuit equipment types, the size of the analog part accounts for approximately 20% of the device size, but the failure probability of the analog part accounts for more than 80% of the failure probability of the entire circuit equipment. Therefore, the analog part of the electronic circuit is a key part of the electronic circuit and therefore the entire system, and in turn critical to the system’s reliability during operation.

Electronic circuit faults are mainly divided into soft faults and hard faults. The hard fault manifests itself as an enormous change in the component parameters. This results in a fundamental change in circuit topology, which greatly damages the overall circuit, such as an open circuit or short circuit. Soft failure refers to the deviation of the relevant parameters of electronic circuit components from typical values. For example, a switching power converter can lose its primary function due to the degradation of the LC filter’s electrolytic capacitors (as would be the case in a Sallen–Key bandpass filter and a four-opamp biquad highpass filter). Therefore, it is clear that the timely diagnosis of soft faults is vital for safe system operation^1^.

Many studies have attempted to solve the key problems of circuit fault detection^2–9^. Although these studies have solved the non-linear problems and tolerance effects of the circuit’s analog part to some extent, they have various disadvantages. Therefore, it is very important to investigate effective and simple feature extraction methods for extracting the basic features of analog circuit faults. To overcome the limitations of traditional methods, this paper proposes a novel analog circuit fault diagnosis method based on deep learning technology and the support vector machine (SVM) optimized by the gray wolf optimization (GWO) algorithm (GWO-SVM).

Analog circuit fault diagnosis is generally divided into two steps: the original signal feature extraction and fault classification. Notably, the deep belief network (DBN) has many advantages contributing to its superior performance in analog circuit fault detection^10–16^. First, the DBN has a multi-layer network structure, and its unique network training method allows the DBN to autonomously extract the features of a target and the essential expression of the data. Secondly, the DBN directly extracts the required features from the original signal without any function mapping and transformation, which can significantly improve the identification or distinguishability of the extracted original signal’s features. Thirdly, the DBN can solve problems regarding the number of label samples and easy localization in training. The SVM model^17,18^ is used in the classification of analog circuit faults. The optimization effect of the SVM largely depends on its penalty factor c, and kernel parameter g. When constructing a diagnostic model, parameters c and g must be set to the optimal values to achieve the best classification effect. The GWO was proposed in 2014^19^ and implements the global optimization process by imitating the gray wolf population hierarchy system and hunting mechanism. The gray wolf optimization algorithm has faster convergence, has more stable operation, and can find suitable parameters for complex nonlinear problems.

This paper proposes an analog circuit fault detection method based on a DBN and GWO-optimized SVM for fault diagnosis. First, the different initial fault modes of the analog circuit are set; then, the DBN is used to extract the circuit’s output signal as a feature. Finally, the GWO-SVM is used to diagnose the fault mode.

## Feature extraction method based on DBN

Deep learning finds the essential expression of data through the layer-by-layer nonlinear mapping expression of the data and then realizes the deep extraction of features. The effective extraction of features is very important for fault diagnosis. The DBN is a typical deep learning method that can effectively extract feature information from the original signal.

### Restricted Boltzmann machine

Support vector machines have the advantages of robustness, good ability for learning from small samples, and strong generalization ability. The restricted Boltzmann machine (RBM) solves the problem of feature division by exploiting high-dimensional space^10,20^.

**Figure 1:**
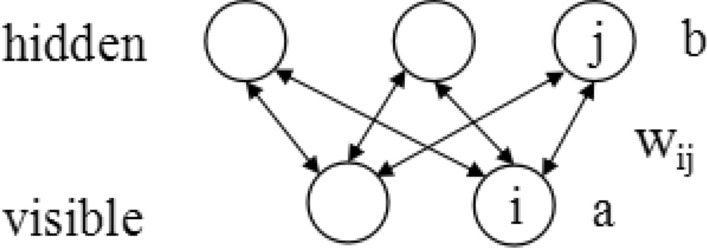
RBM structure.

The RBM model is shown in Fig. 1. There is a bidirectional connection between each unit of each layer, but no connection between the units in the layer. The input vector corresponds to the visible vector of the RBM. The hidden vector is the feature extracted from the visible vector. The energy of the RBM unit configuration is defined as follows:

1 $$E\left(v,h\right)=-{\sum }_{i=1}^{I}{v}_{i}{a}_{i}-{\sum }_{j=1}^{J}{b}_{j}{h}_{j}-{\sum }_{i=1}^{I}{\sum }_{j=1}^{J}{h}_{i}{v}_{i}{w}_{ij}$$

where W_ij_ is the connection weight of the visible layer unit i and the hidden layer unit j; a_i_ is the offset of the visible layer unit i; bj is the offset of the hidden layer unit j.

The conditional distribution of the visible layer is expressed as follows:

2 $$P\left({h}_{j}=1\left|v\right.\right)=\sigma \left({\sum }_{i}{w}_{ij}{v}_{i}+{b}_{j}\right)$$

The conditional distribution of the hidden layer is expressed as follows:

3 $$P\left({v}_{i}=1\left|h\right.\right)=\sigma \left({\sum }_{j}{w}_{ij}{h}_{j}+{a}_{i}\right)$$

where $\sigma$ generally uses the sigmoid function as the activation function.

The joint probability distribution between the visible layer and the hidden layer is defined as follows:

4 $$P\left(v,h\right)=\frac{1}{Z}{e}^{-E\left(v,h\right)}$$

where Z is obtained by summing all possible visible and hidden vectors, as follows:

5 $$Z={\sum }_{v}{\sum }_{h}{e}^{-E\left(v,h\right)}$$

When inputting v, the hidden layer neuron h can be calculated using (2). Once the hidden layer is obtained, the reconstruction of the visible layer can be obtained using (3). When each v_i_ in (3) is set to 1, the visible layer can be reconstructed.

### Feature extraction based on DBN

**Figure 2:**
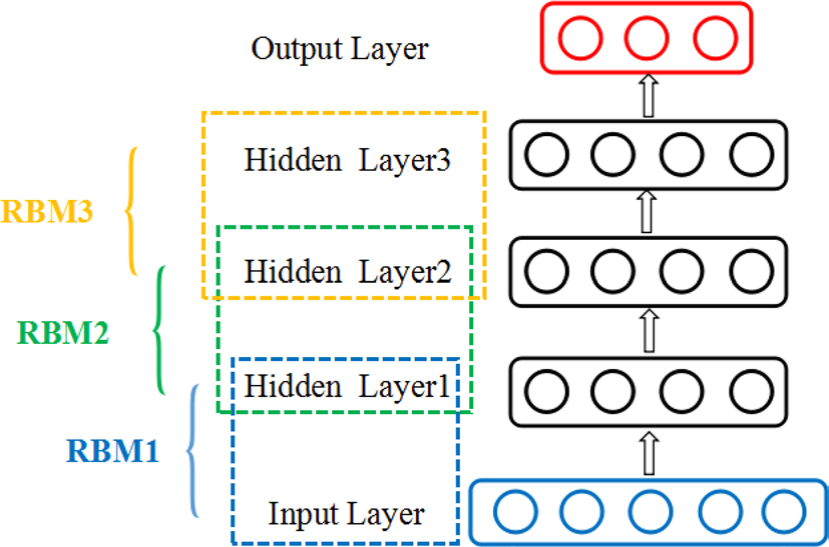
Three-layer DBN structure.

The DBN training process is essentially the process of mining deep representation information from the original features. The DBN is formed by stacking several RBMs, and the training process is performed layer by layer from low to high. Figure 2 shows a DBN consisting of three hidden layers.

6 $$\Delta {w}_{ij}=\varepsilon \left({\langle {v}_{i}{h}_{j}\rangle }_{P\left(h\left|v\right.\right)}-{\langle {v}_{i}{h}_{j}\rangle }_{recon}\right)$$

7 $$\Delta {b}_{j}=\varepsilon \left({\langle {h}_{j}\rangle }_{P\left(h\left|v\right.\right)}-{\langle {h}_{j}\rangle }_{recon}\right)$$

8 $$\Delta {a}_{i}=\varepsilon \left({\langle {v}_{i}\rangle }_{P\left(h\left|v\right.\right)}-{\langle {v}_{i}\rangle }_{recon}\right)$$

where $\varepsilon$ represents the learning rate, and ${\langle\cdot \rangle }_{\mathrm{recon}}$ represents the expectation of the partial derivative under the reconstructed model distribution.

In the fine-tuning phase, the back-propagation algorithm is used to train the DBN network in reverse by fine-tuning the weight of each connection to improve the feature extraction effect and reduce the diagnostic error. The overall fine-tuning phase is a supervised learning process. The feature extracted by the last RBM after fine tuning is the feature extracted by the DBN.

## Classifier based on GWO-SVM

When the pre-training and fine-tuning stages of the DBN are completed, it is necessary to accurately realize the fault division and location of the circuit. This study used the support vector machine algorithm to diagnose the faulty component.

### Support vector machine

The SVM constructs the best separation hyperplane in high-dimensional space to separate the decision categories. If the following conditions are satisfied, the training mode can be separated, as follows:

9 $$ y_{i} \left( {w \cdot x_{i} + b} \right) \ge 1 $$

where w is the normal vector of the hyperplane and b is the offset of the hyperplane.

Using the given training set data for training, the optimal solutions w* and b* of the hyperplane separated by the maximum interval can be determined as follows:

10 $$\underset{w,b}{min}\frac{1}{2}{\Vert w\Vert }^{2}$$

The classification decision function is expressed as follows:

11 $$f\left(x\right)=sign\left({w}^{*}\cdot x+{b}^{*}\right)$$

In the case of non-linearly separable samples, a kernel function is typically used to construct the classifier. By using kernel functions, samples can be mapped to high-dimensional space to solve linearly inseparable problems in low-dimensional space. By introducing the relaxation variable ξi and the penalty function C, the problem becomes as follows:

12 $$\underset{w,b,\xi }{min}\frac{1}{2}{\Vert w\Vert }^{2}+C{\sum }_{i=1}^{N}{\xi }_{i}$$

where N is the number of samples, i = 1, 2, … N.

In this case, the classification decision function is expressed as follows:

13 $$f\left(x\right)=sign\left(C{\sum }_{i=1}^{N}{\xi }_{i}+\frac{1}{2}\Vert w\Vert \right)$$

In the case of linear inseparability, the input is mapped to a high-dimensional space, the kernel function K (x, xi) is introduced, and the linear classification after the nonlinear transformation is realized using the kernel function. The SVM optimal classification decision function is expressed as follows:

14 $$f\left(x\right)=sign\left({\sum }_{i=1}^{N}{{a}_{i}}^{*}{y}_{i}K\left(x\cdot {x}_{i}\right)+{b}^{*}\right)$$

where K(x, y) represents the kernel function. In this study, the radial basis function (RBF) was adopted.

Because the classification accuracy of the SVM model is greatly influenced by the parameter combination penalty factor c and the value of the kernel function kernel parameter g, this study used the GWO algorithm to optimize the selection of the optimal parameter combination of the SVM model.

### GWO optimizes SVM

The gray wolf optimization algorithm imitates the species leaderships and hunting mechanisms of gray wolves^19^, who surround, hunt, and attack according to their social rank. The predation tasks are assigned to different gray wolf groups to complete the predation behavior, so as to achieve global optimization. Compared with particle swarm optimization, genetic algorithm, grid search, and so on, the gray wolf algorithm has the advantages of having simple structure, easy operation, and short optimization time. In the GWO algorithm, the wolf with the strongest leadership ability is α, and is mainly responsible for the decision-making part of the optimization process; the remaining gray wolves are sequentially recorded as β, δ, and ω. Alpha is the optimal solution, β and δ are the sub-optimal solutions, and ω is the lowest gray wolf.

The distance between the prey and the wolves is expressed as follows:

15 $$D=\left|C\cdot {X}_{P}\left(t\right)-X\left(t\right)\right|$$

16 $$X\left(t+1\right)={X}_{P}\left(t\right)-A\cdot D$$

where t is the number of iterations; Xp(t) is the position of the prey after the tth iteration, that is, the position of the optimal solution; X(t) is the position of the gray wolf after the tth iteration, that is, the position of the potential solution; A and C are coefficient factors, and can be calculated as follows:

17 $$A=2a\cdot {r}_{1}-a$$

18 $$C=2\cdot {r}_{2}$$

where the value of a linearly decreases from 2 to 0 as the number of iterations increases; r1 and r2 are random numbers between [0, 1]. The mathematical model of gray wolf rounding behavior is expressed as follows:

19 $$ \begin{aligned} D_{\alpha } & = \left| {C_{1} X_{\alpha } \left( t \right) - X\left( t \right)} \right| \\ D_{\beta } & = \left| {C_{2} X_{\beta } \left( t \right) - X\left( t \right)} \right| \\ D_{\delta } & = \left| {C_{3} X_{\delta } \left( t \right) - X\left( t \right)} \right| \\ \end{aligned} $$

20 $$ \begin{aligned} X_{1} & = X_{\alpha } \left( t \right) - A_{1} D_{\alpha } \\ X_{2} & = X_{\beta } \left( t \right) - A_{2} D_{\beta } \\ X_{3} & = X_{\delta } \left( t \right) - A_{3} D_{\delta } \\ \end{aligned} $$

21 $${X}_{P}\left(t+1\right)=\frac{\left({X}_{1}+{X}_{2}+{X}_{3}\right)}{3}$$

where $D_{\partial }$, $D_{\beta }$, $D_{\delta }$ and represent the distance between the α, β, δ, and ω wolves (that is, other individuals). Equations (19), (20), and (21) ensure that the gray wolf population obtains a better position than the initial point. When the optimal position appears, Xp is updated to a better position.

**Figure 3:**
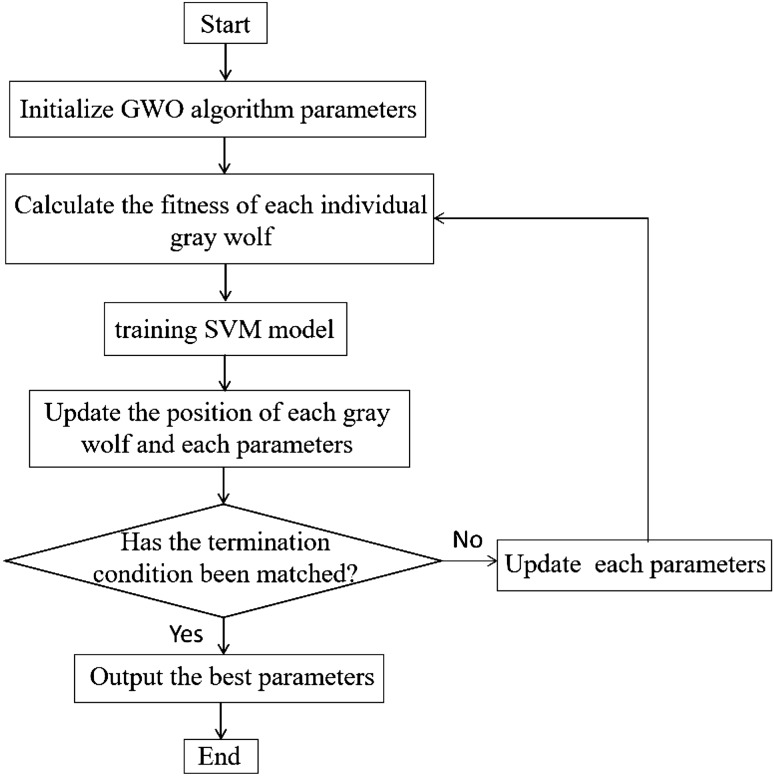
Flowchart of SVM optimized by GWO algorithm.

The flow chart of GWO optimization SVM is shown in Fig. 3:

The optimization process of SWO optimized by GWO is:

*Step 1* Initialize parameters such as population size N, distance D, maximum iteration number t, and randomly generated parameters a, A, and C;

*Step 2* Calculate the fitness value of each gray wolf individual, record the position of the top three gray wolf individuals as Xa, Xb, Xc, and record the best fitness Xa as the optimal solution at this time;

*Step 3* Train the SVM model, the best parameters c, g are the output of two Xa;

*Step 4* Calculate the distance between the remaining individuals ω and Xa, Xb, Xc according to Eq. (19), and update the position of the gray wolf α, β, δ and prey;

*Step 5* Update the values of parameters a, A, and C according to Eqs. (17) and (18);

*Step 6* If the algorithm reaches the maximum number of iterations t, then the algorithm ends and outputs the optimal solution Xa; otherwise, update the parameter values of each part and return to step2.

## Simulation experiments and analysis

### Analog circuit fault definition

**Figure 4:**
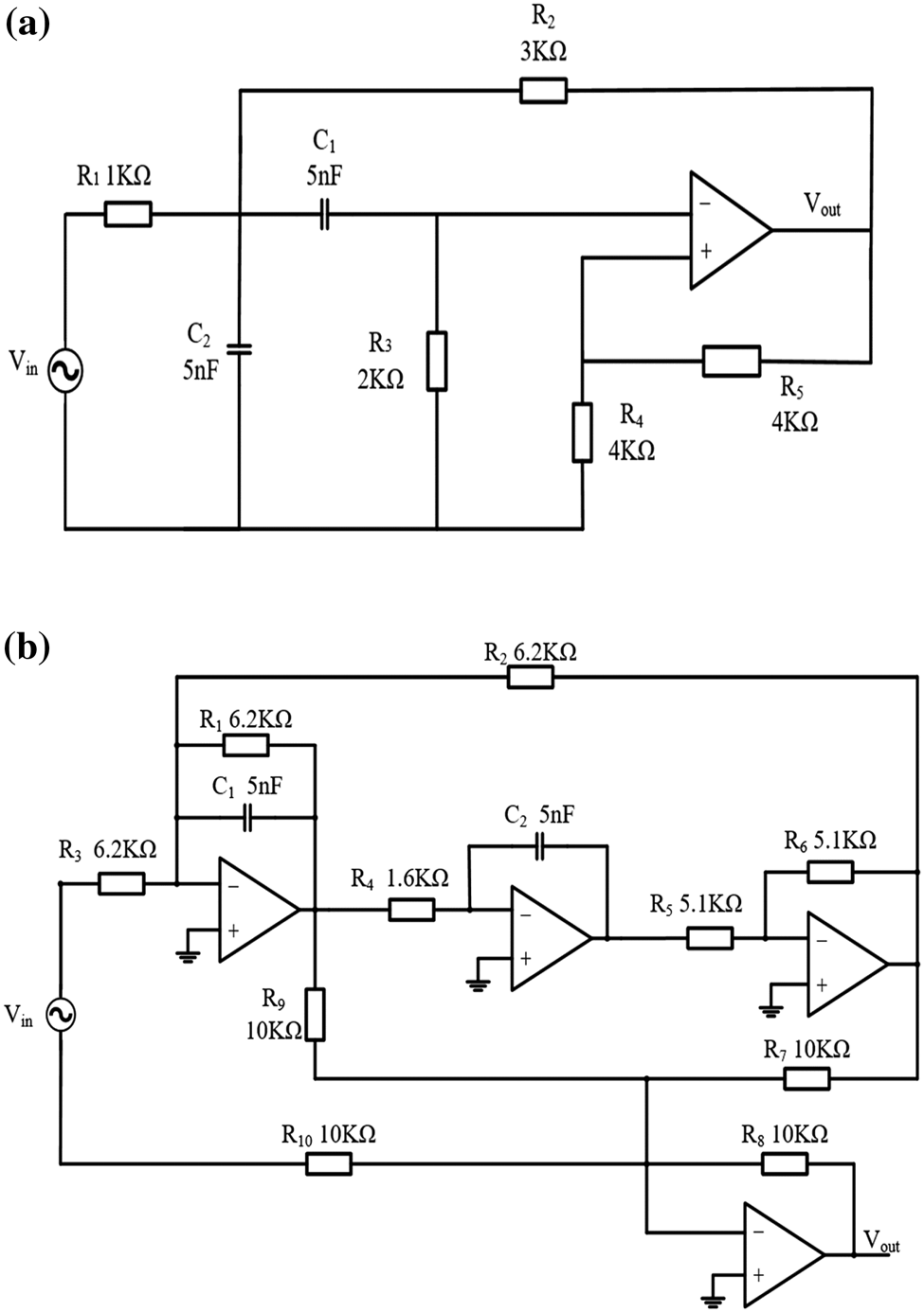
(**a**) Schematic of Sallen–Key bandpass filter circuit (Case 1); (**b**) Schematic of four-op-amp biquad highpass filter circuit (Case 2).

Active filters are widely used by in many electronic circuits used for example in power systems, control systems, utility systems and used by precision electronics companies, medical institutions, and so on. Therefore, active filters play a key role in large electronic equipment systems. However, in practical applications, filter faults destroy system functions and even cause catastrophic losses. Therefore, in this study, the Sallen–Key band-pass filter circuit and a four-opamp biquad highpass filter circuit were selected as the test circuits, as shown in Fig. 4.

For the Sallen–Key bandpass filter circuit in Fig. 4a, after sensitivity analysis^16^, the center frequency of the circuit is more affected by R2, R3, C1, and C2; therefore, R2, R3, C1, and C2 are used as experimental components. The same reason, C1, C2, R1, R2, R3, R4 are used as experimental components for four-op-amp biquad highpass filter circuit as shown in Fig. 4b. Because the frequency of a single device fault in the filter circuit is higher than the frequency of multiple device failures, this study only investigated the case of a single device fault^21^.

The first case investigated in this study is the Sallen–Key band-pass filter shown in Fig. 4a (hereinafter referred to as Case 1); the nominal values of the components are shown in Fig. 4a. Assuming that only one of the R2, R3, C1, and C2 components fails in each test, the circuit forms the nine different fault categories listed in Table 1 that including no fault mode, where ↑ and ↓ indicate a value higher or lower than the nominal value, respectively, and the tolerance values of the resistance and capacitance are 5% and 10%, respectively. Similarly, the fault categories of the four-op-amp biquad highpass filter circuit (hereinafter referred to as Case 2) are listed in Table 2.

**Table 1:** Fault mode and fault value of Sallen key bandpass filter circuit.

Fault mode	Fault class	Normal value	Fault value
F0	Normal	–	–
F1	C1↓	5 nF	2.5 nF
F2	C1↑	5 nF	10 nF
F3	C2↓	5 nF	2.5 nF
F4	C2↑	5 nF	10 nF
F5	R2↓	3 kΩ	1.5 kΩ
F6	R2↑	3 kΩ	6 kΩ
F7	R3↓	2 kΩ	1 kΩ
F8	R3↑	2 kΩ	4 kΩ

**Table 2:** Fault mode and fault value of four-op-amp biquad highpass filter circuit.

Fault mode	Fault class	Normal value	Fault value
F0	Normal	–	–
F1	C1↑	5 nF	10 nF
F2	C1↓	5 nF	2.5 nF
F3	C2↑	5 nF	15 nF
F4	C2↓	5 nF	2.5 nF
F5	R1↑	6.2 KΩ	15 kΩ
F6	R1↓	6.2 kΩ	3 kΩ
F7	R2↑	6.2 kΩ	18 kΩ
F8	R2↓	6.2 kΩ	2 kΩ
F9	R3↑	6.2 kΩ	12 kΩ
F10	R3↓	6.2 kΩ	27 kΩ
F11	R4↑	1.6 kΩ	25 kΩ
F12	R4↓	1.6 kΩ	500 Ω

### Feature extraction and fault classification

**Figure 5:**
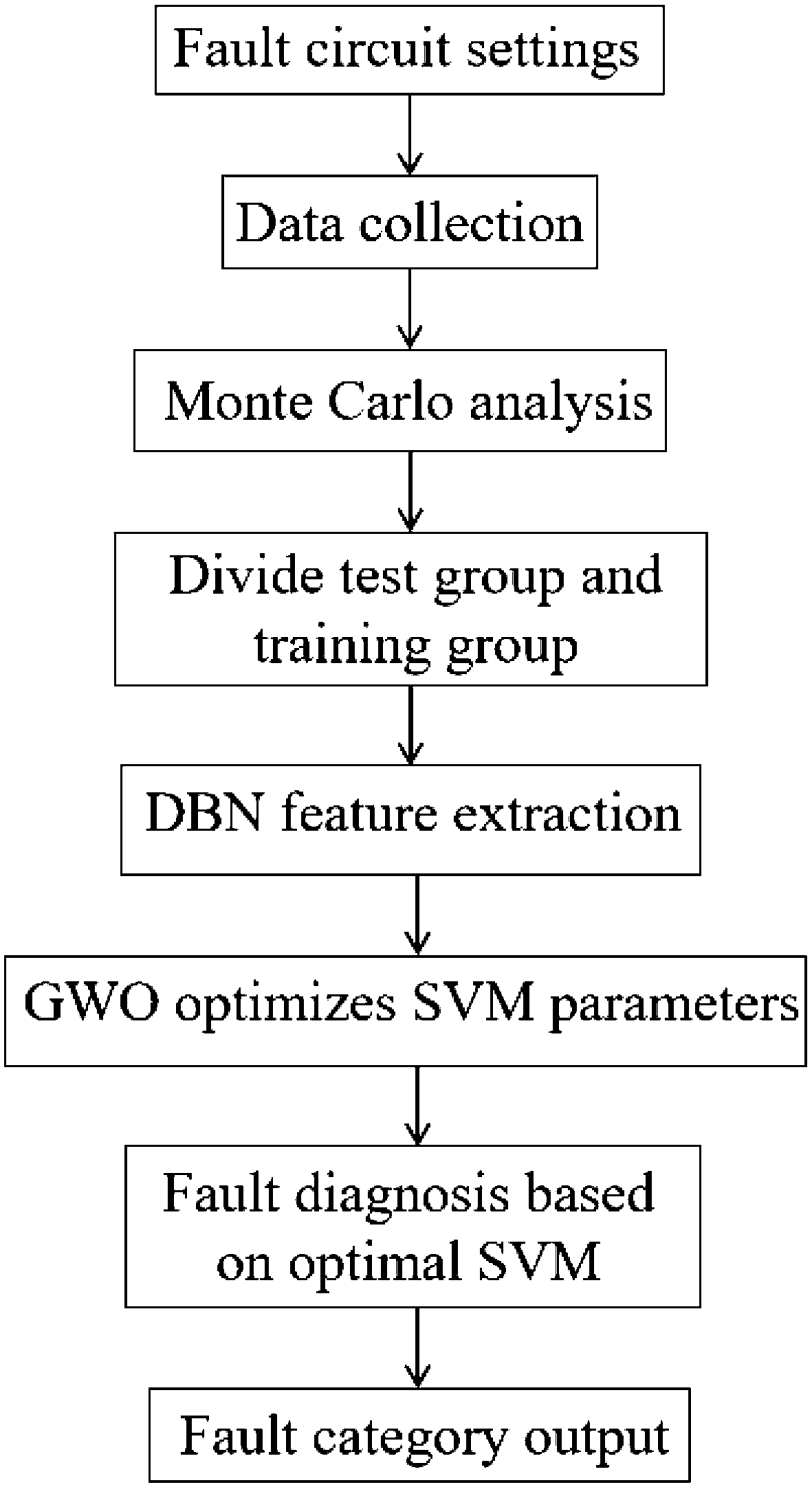
Flowchart of feature extraction and fault classification.

The proposed fault detection method for analog circuits is described as follows: first, use the DBN model to extract the features of the analog circuit’s output data. Then, use the GWO-optimized SVM classifier to classify the fault model. The specific method includes the steps shown in Fig. 5.

*Step 1* Set the fault circuit mode and tolerance value of the faulty device. Case 1 has nine different fault categories, and Case 2 has 13 different fault categories.

*Step 2* Use time-domain transient analysis to collect the signal information output of the simulation in step 1 and save it by category.

*Step 3* Use Monte Carlo analysis to obtain 240 instances of each fault category.

*Step 4* Divide the data obtained in step 3 into a training group and a test group.

*Step 5* use the DBN to extract the features of the training group data.

*Step 6* Use the GWO optimization algorithm to optimize the c and g parameters of the SVM.

*Step 7* Perform fault diagnosis based on the feature data extracted by the DBN and optimal SVM model.

*Step 8* Output the fault diagnosis result.

The PSPICE software was used for data generation in the experiment, and the output amplitude-frequency characteristic curve of the circuit was obtained through AC analysis. The peak voltage of the circuit was 10 V, and a sinusoidal voltage source with a frequency of 1 kHz was selected as the excitation. Within the duration of 0 -1.2 ms, 128 data points were output in each group mode of the output node. Then Monte Carlo analysis was carried out to obtain 240 instances of each fault category. The total number for Case 1 was 2160, and the total number of examples for Case 2 was 3120. In the experiment, a DBN with three hidden layers was used.

**Figure 6:**
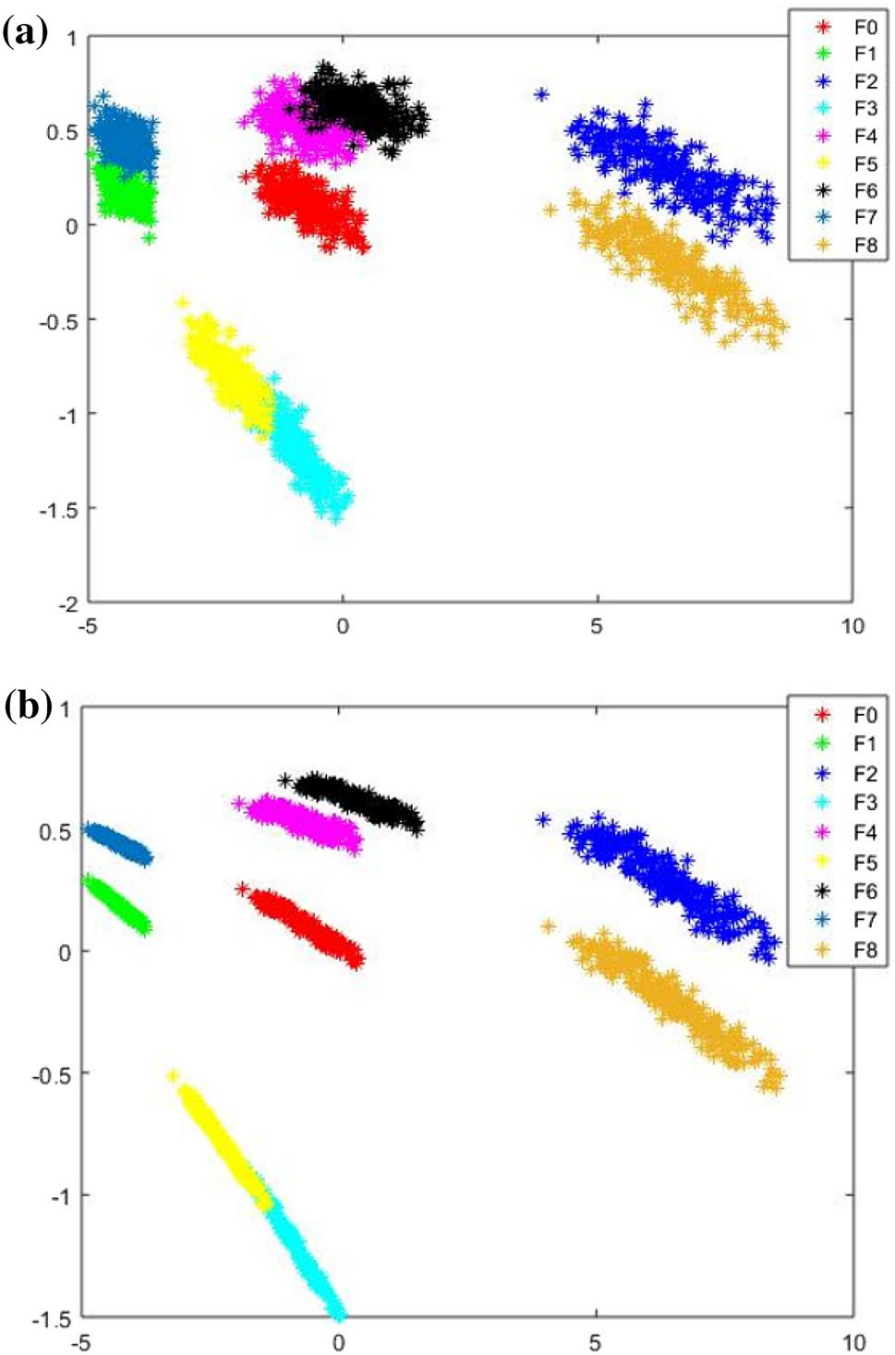
Characteristic distribution diagram of Case 2: (**a**) row data; (**b**) data after DBN feature extraction.

For Case 1, the circuit diagram is shown in Fig. 4a, and the DBN structure was set to 128-64-32-16-9. Based on experience^22,23^, the number of pre-training iterations for each RBM was 100, the number of fine-tuning iterations was 500, and the learning rate and momentum were set to 0.11 and 0.9, respectively. To clearly demonstrate the superior performance of the DBN method in feature extraction, this study used the kernel principal component analysis method (KPCA) to reduce the dimension of the original data and feature data extracted by the DBN. Then, the data features in the two cases were visualized in the spatial distribution. Figure 6a is a visual feature distribution diagram of the original data in Case 1 after dimensionality reduction. Figure 6b shows the visual feature distribution diagram of the original data of Case 1 after the DBN extracted the features. Compared with the original data visualization scatter diagram, there is only a slight overlap, and approximately all remaining modes are separated.

**Figure 7:**
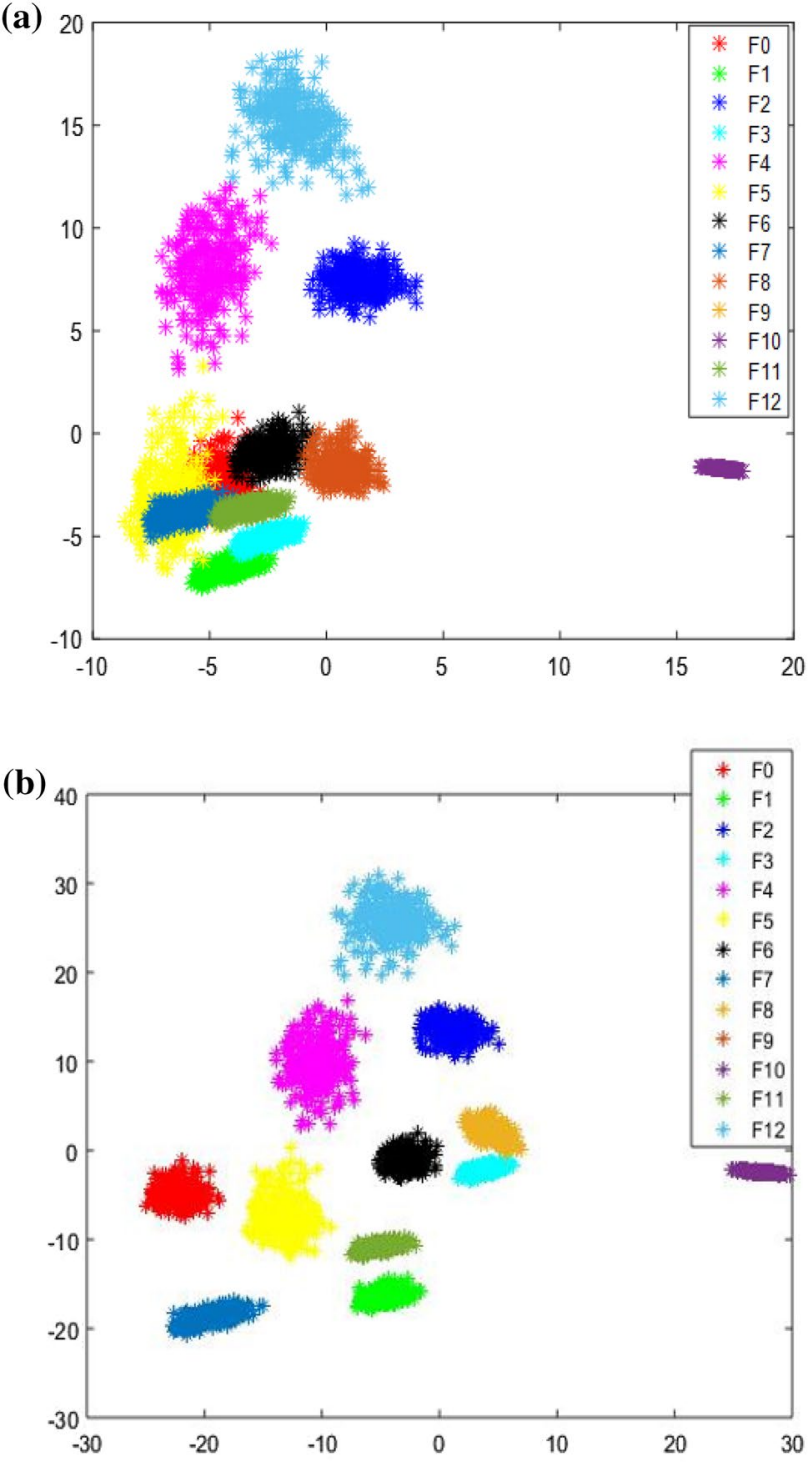
Characteristic distribution diagram of Case 2: (**a**) row data; (**b**) data after DBN feature extraction.

In Case 2, the DBN structure was set to 128-64-32-16-13. The remaining parameter settings were the same as in Case 1. Figure 7a shows the visual scatter diagram of the original data in Case 2, and Fig. 7b shows the visual scatter diagram of the original data after the DBN extracted the features. Similar to Case 1, the different failure modes in the original data have severe overlaps. After the DBN extracted the features the overlapping parts were largely separated, as shown in Fig. 7b.

Based on the effective feature information extracted by the DBN, the GWO-SVM diagnostic model is used to classify the fault category. To improve the classification accuracy and shorten the classification time of the SVM, the GWO optimization algorithm is used to optimize the penalty coefficient c and kernel function parameter g of the SVM. In this study, the appropriate population size was set to 10, and the number of iterations was set to 100. The best parameters in Case 1 and Case 2 are listed in Table 3. For Case 1, when c was 93.3724 (retaining four decimal places) and g was 3.7042, the parameter combination of the GWO-SVM achieved the best effect, and the classification accuracy of Case 1 was 100%. For Case 2, when c was 100 and g was 26.9118, the GWO-SVM parameter combination achieved the optimal effect, and the case classification accuracy of Case 2 was 99.68% (retaining two decimal places).

**Table 3:** Best parameters and accuracy in Case 1 and Case 2.

Category	Best c parameter	Best g parameter	Accuracy
Case 1	93.3724	3.7042	100%
Case 2	100	26.9118	99.68%

### Analysis of fault classification result

**Figure 8:**
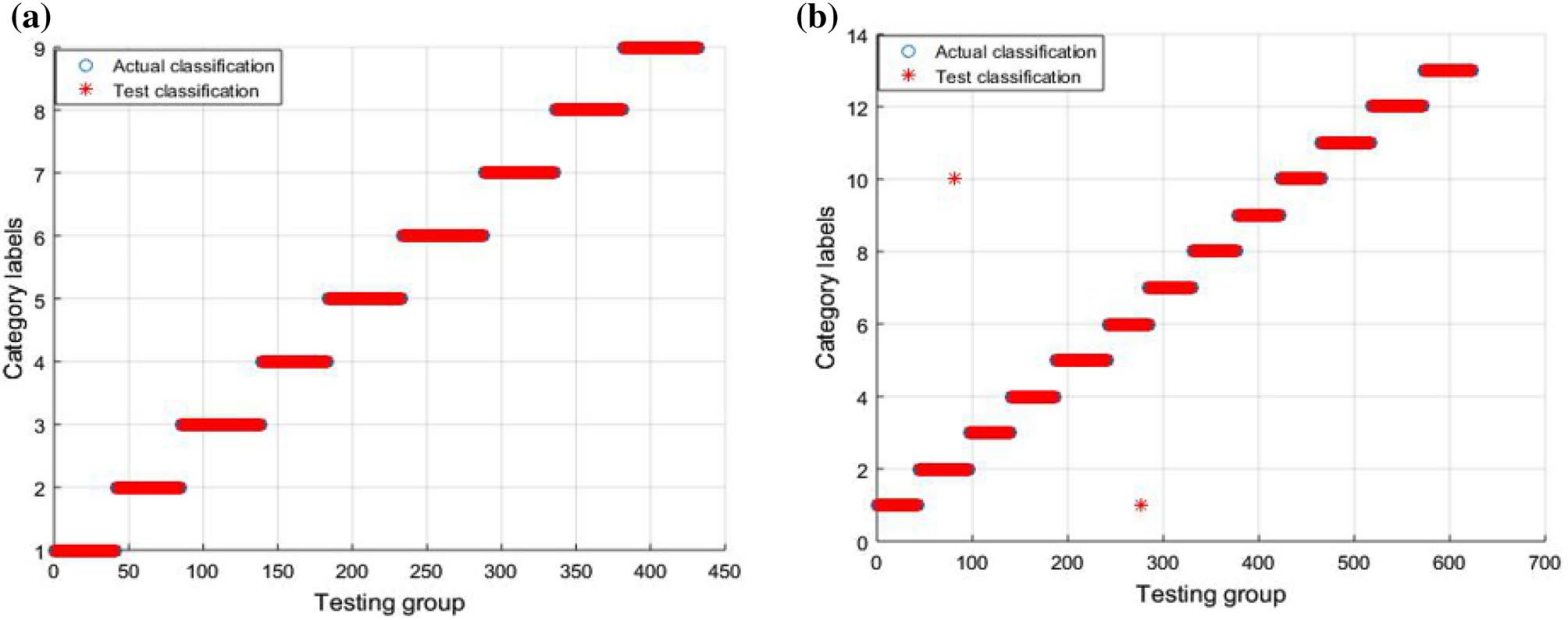
Fault classification result in GWO-SVM: (**a**) classification result for Case 1; (**b**) classification result for Case 2.

For the experiments described in this section, we propose a diagnostic method for extracting data features from the DBN and classifying the faults using GWO-SVM. The proposed fault diagnosis method achieved diagnosis accuracy of 100% for the classification of circuit faults in Case 1, and diagnosis accuracy of 99.68% for the classification of circuit faults in Case 2. The circuit fault classification result is presented in Fig. 8.

**Figure 9:**
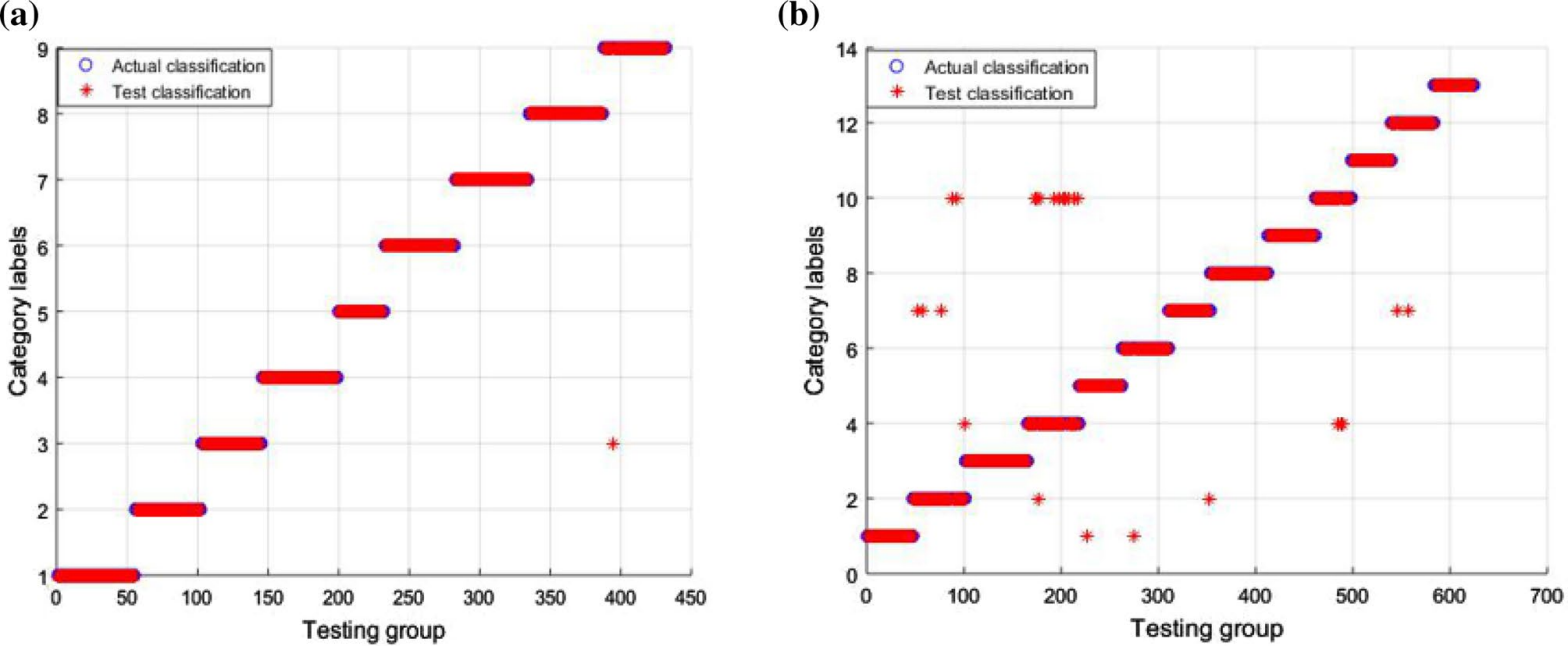
Fault classification result in GA-SVM: (**a**) classification result for Case 1; (**b**) classification result for Case 2.

**Figure 10:**
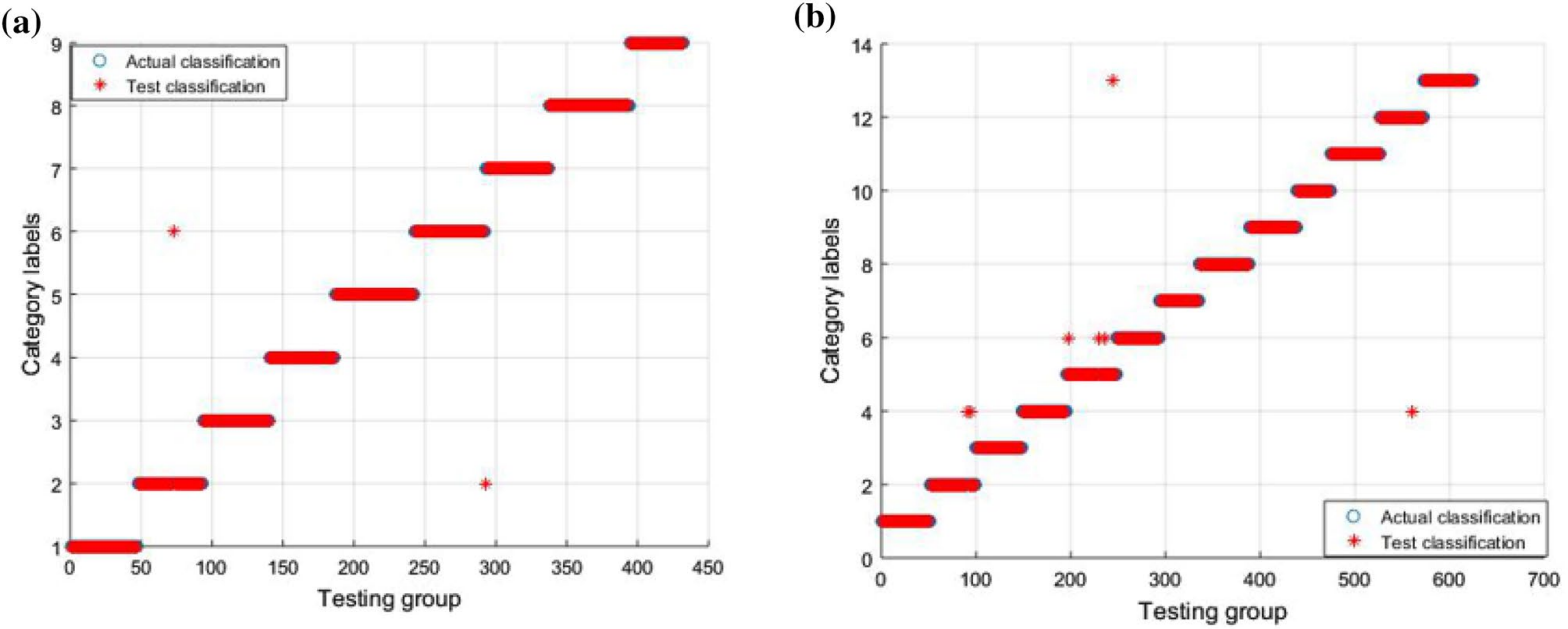
Fault classification result in PSO-SVM: (**a**) classification result for Case 1; (**b**) classification result for Case 2.

In Fig. 8a, it can be seen that the fault classification in Case 1 is entirely correct. In Fig. 8b, it can be seen that, for the fault classification in Case 2, only the first and tenth categories include erroneous data. To verify that the GWO-SVM has faster convergence speed and higher accuracy, the GWO-SVM was compared with the GA-SVM and PSO-SVM. For fault classification, the fault features extracted by the DBN were input into the GWO-SVM, GA-SVM, and PSO-SVM, respectively. The fault diagnosis results of GA-SVM and PSO-SVM are shown in the Fig. 9 and Fig. 10. Obviously, compared with GWO-SVM, the fault diagnosis accuracy of GA-SVM and PSO-SVM for the two circuits is significantly reduced.

**Figure 11:**
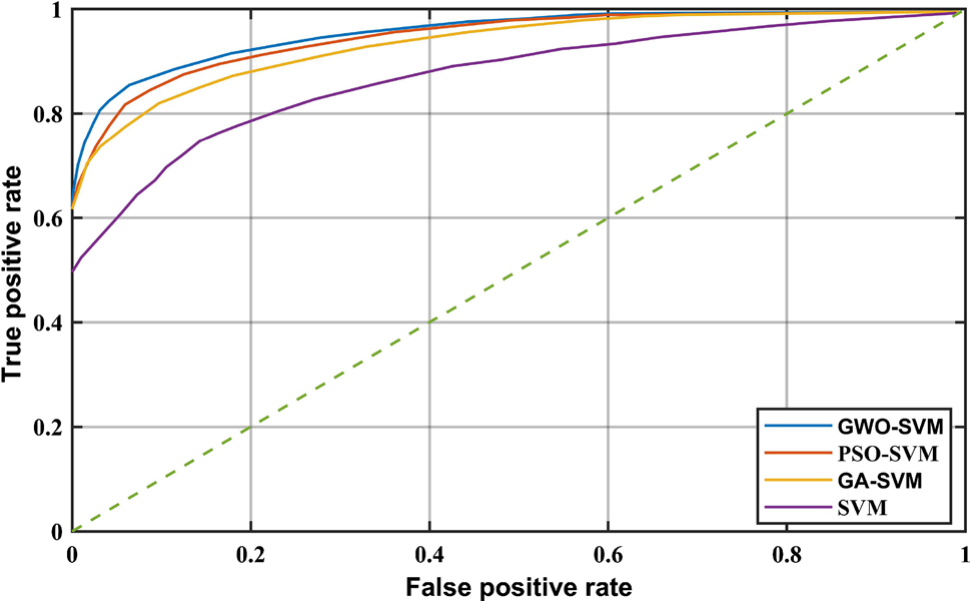
ROC curve of four classifiers.

Receiver operating characteristic curve (ROC) can represent the trend of the sensitivity and accuracy of the classifier. The closer the curve is to the upper left corner, the better the performance of the classifier is. Generally, the ROC curve of the classifier is drawn to analyze its performance. By comparing GWO-SVM, GA-SVM, PSO-SVM and original SVM, the ROC curve is shown in Fig. 11. It can be seen from the figure that the ROC curve of GWO-SVM is closest to the upper left corner, which indicates that GWO-SVM has better performance.

**Figure 12:**
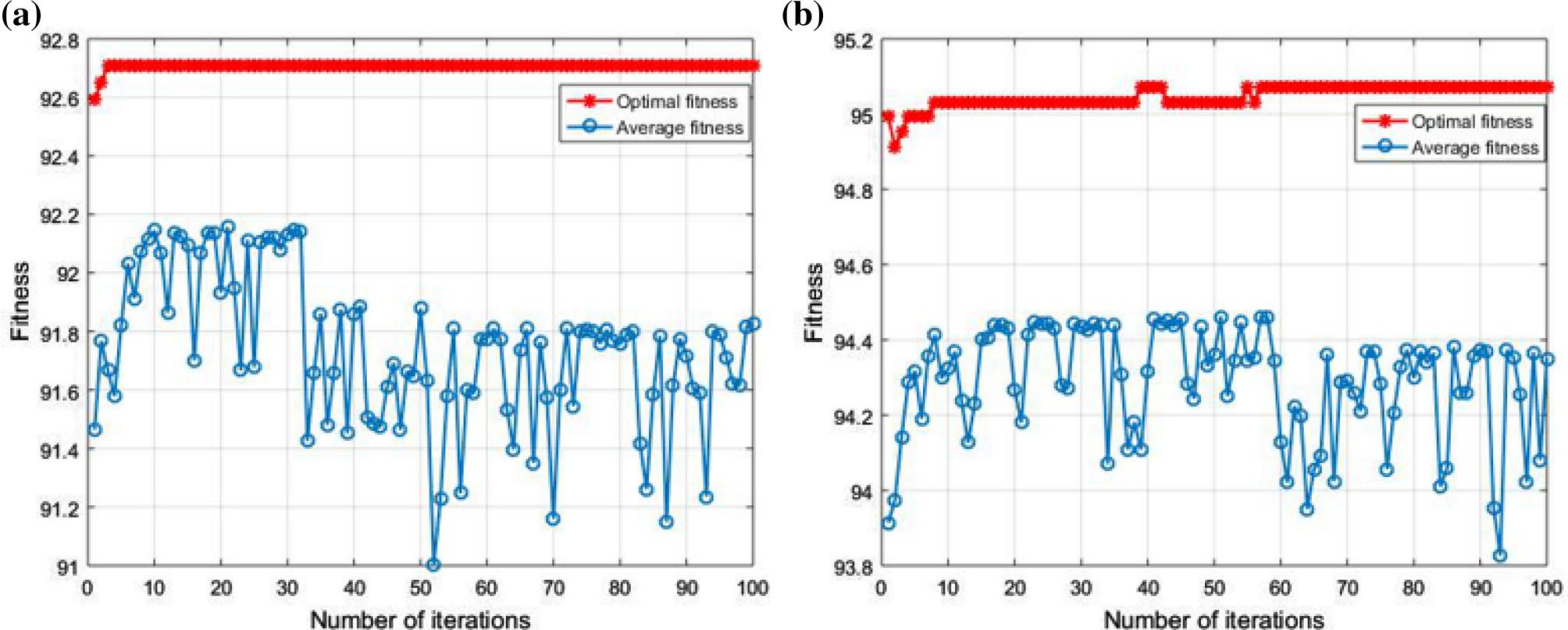
GA-SVM fitness curve: (**a**) fitness curve for Case 1; (**b**) fitness curve for Case 2.

**Figure 13:**
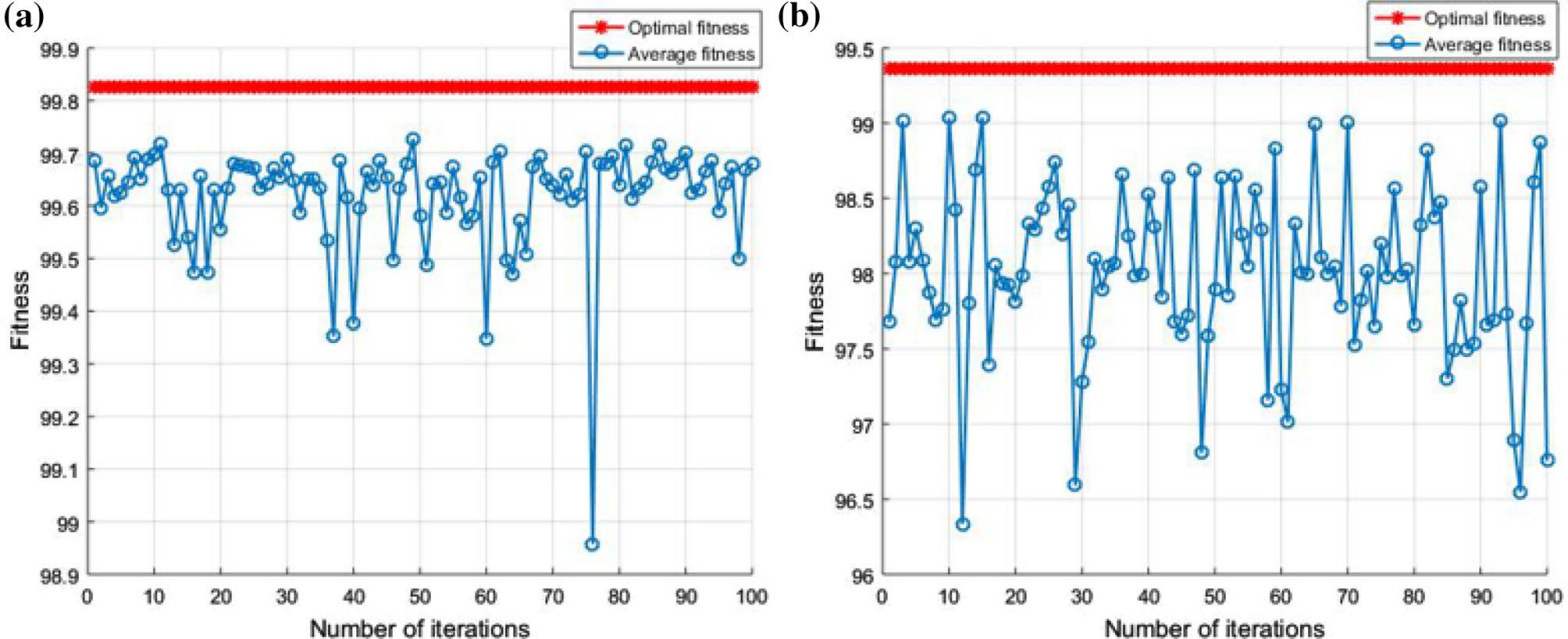
PSO-SVM fitness curve: (**a**) fitness curve for Case 1; (**b**) fitness curve for Case 2.

**Figure 14:**
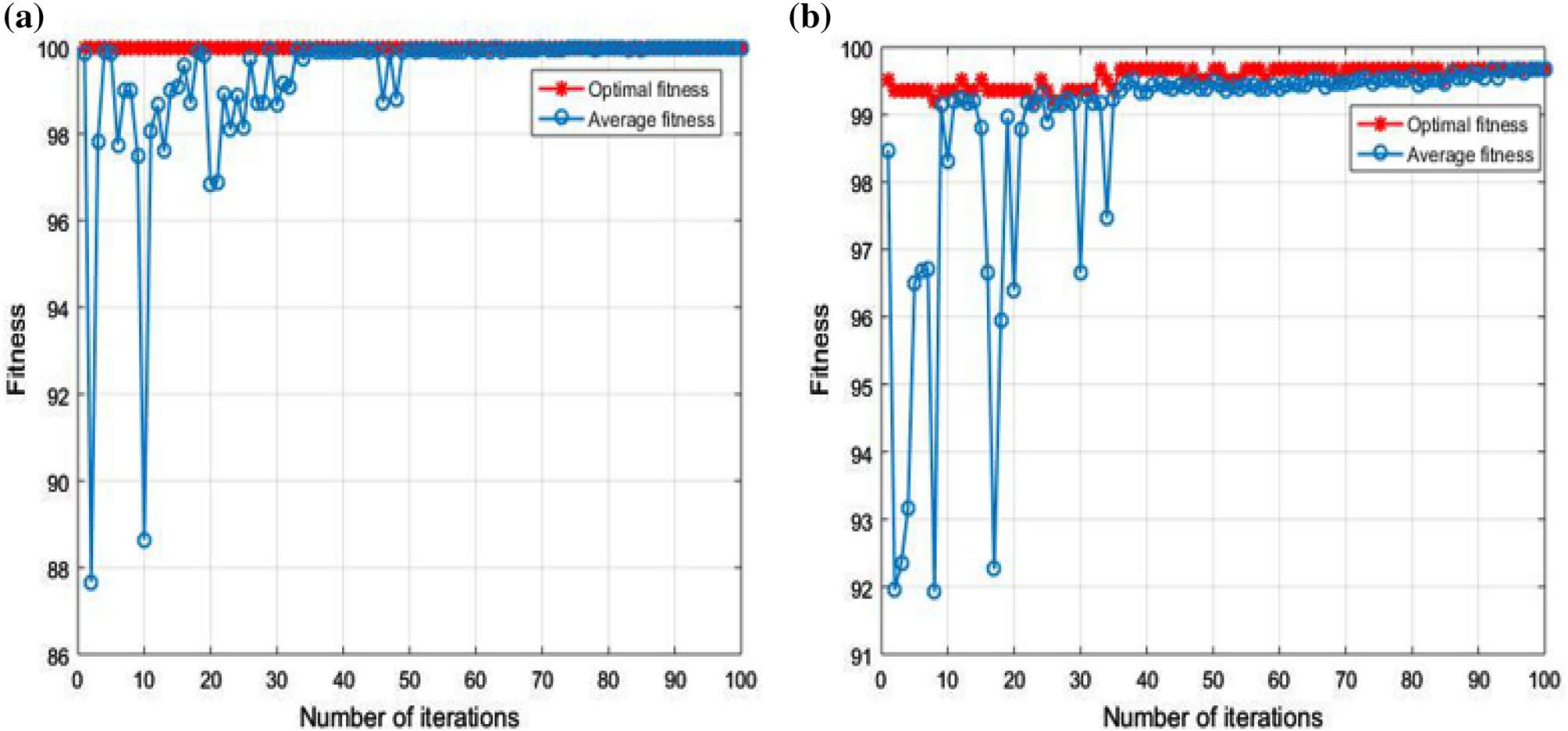
GWO-SVM fitness curve: (**a**) fitness curve for Case 1; (**b**) fitness curve for Case 2.

The diagnostic performance of the three different classifiers in the two cases is presented in Tables 4 and 5. By comparison, it can be seen that the performance of GWO-SVM is superior to that of the other three classifiers. The test accuracy of GWO-SVM has a higher value. In Case 1, the classification accuracy of GWO-SVM was 100%, and the optimization process was completed in 18.48 s, the fault diagnosis time has been shortened by approximately 94%. In Case 2, the classification accuracy of GWO-SVM was 99.68%, and the optimization process was completed in 60.07 s, the fault diagnosis time has been shortened by approximately 91%. Figure 12 shows the fitness curves of the GA-SVM, Fig. 13 shows the fitness curves of the PSO-SVM, and Fig. 14 shows the fitness curves of the GWO-SVM. From the three figures, it can be seen that GWO-SVM classification has the highest accuracy and fastest convergence rate. Compared with other classifier models, the proposed GWO-SVM model only required one iteration in Case 1 to achieve the best fitness. In Case 2, the model only required 10 iterations to achieve the best fitness and stabilized after 35 iterations, compared with other models, the proposed model reduces hundreds of iterations and saves a lot of time. The results reveal that the proposed fault diagnosis method does not only significantly improve the convergence speed, but also has a low prediction error and achieves more robust prediction results.

**Table 4:** Comparison table of results obtained by three optimization algorithms for Sallen–Key bandpass filter.

Classification model	Best c parameter	Best g parameter	Accuracy	Time (s)
GA-SVM	91.9356	952.224	92.82%	333.75
PSO-SVM	326.439	28.4965	99.54%	336.90
GWO-SVM	93.3724	3.7042	100%	18.48

**Table 5:** Comparison table of results obtained by three optimization algorithms for four-opamp biquad highpass filter.

Classification model	Best c parameter	Best g parameter	Accuracy	Time (s)
GA-SVM	85.596	965.37	94.87%	659.06
PSO-SVM	645.788	6.6136	98.87%	814.71
GWO-SVM	100	26.9118	99.68%	60.07

Moreover, we compare the proposed method with traditional analog circuit fault diagnosis methods and new technologies^5,14,16,23–25^. As shown in Table 6, using the optimized classification tool can improve the classification performance. In addition, compared with reference^25^, the proposed method not only improves the accuracy of fault diagnosis, but also reduces the fault diagnosis time of the two cases by 89.98% and 74.67% respectively. In a word, the feature extraction method based on Dynamic Bayesian network proposed in this paper can learn deep and intrinsic data features from the original data, besides, GWO-SVM improves the performance of the classifier. Compared with the previous methods, the proposed method obtains the highest diagnostic accuracy and the shortest fault diagnosis time.

**Table 6:** Comparison of fault diagnosis accuracy of proposed method to accuracy achieved by previously proposed methods.

Method	Case 1 (%)	Case 2 (%)
Method in^5^	99.07	92.95
Method in^14^	98.09	99.43
Method in^16^	100	95.51
Method in^23^	100	95.90
Method in^24^	94.16	97.14
Method in^25^	98.33	98.32
Proposed method	100	99.68

## Conclusions

This paper proposes a fault diagnosis method based on DBN feature extraction and GWO-SVM classification. Different from traditional feature extraction methods, DBN can perform unsupervised deep feature learning to form hierarchical model. Additionally, the use of the GWO-optimized SVM greatly improves the accuracy of SVM classification and shortens the working time of the SVM. The results reveal that the GWO-SVM method can reduce hundreds of iterations and shorten calculation time by more than 75%. Although the proposed analog circuit fault diagnosis method has higher diagnosis accuracy compared with traditional diagnosis methods, the disadvantage of this method is that significantly large data sets will reduce the diagnostic performance. For large circuits, we guess that the circuit with complex structure can be divided into many different parts, and the output signal of each different module can be used for fault diagnosis, so as to realize the fault diagnosis of the whole circuit. Future work will focus on using analog circuit fault diagnosis theory in more complex circuits and in practical circuit fault diagnosis.

## Data Availability

All data, models, and code generated or used during the study appear in the submitted article.

## References

[1] Singh B, Al-Haddad K, Chandra A (1999). A review of active filters for power quality improvement. IEEE Trans. Ind. Electron..

[2] Aminian F, Aminian M (2001). Fault diagnosis of analog circuits using Bayesian neural networks with wavelet transform as preprocessor. J. Electron. Test..

[3] Aminian M, Aminian F (2007). A modular fault-diagnostic system for analog electronic circuits using neural networks with wavelet transform as a preprocessor. IEEE Trans. Instrum. Meas..

[4] Aminian M, Aminian F (2000). Neural-network based analog-circuit fault diagnosis using wavelet transform as preprocessor. IEEE Trans. Circuits Syst. II Analog Digit. Signal Process..

[5] Aminian F, Aminian M, Collins HW (2002). Analog fault diagnosis of actual circuits using neural networks. IEEE Trans. Instrum. Meas..

[6] Halder, A. & Chatterjee, A. Automated test generation and test point selection for specification test of analog circuits. In *Proc. 5th Int. Symp. Quality Electron. Des*. 401–406 (2004).

[7] Grzechca, D., Rutkowski, J. & Golonek, T. PCA application to frequency reduction for fault diagnosis in analog and mixed electronic circuit. In *Proc. Int Symp. Circ. Syst. (ISCAS)*. 1919–1922, (IEEE , 2010).

[8] Yuan L, He Y, Huang J, Sun Y (2010). A new neural-network-based fault diagnosis approach for analog circuits by using kurtosis and entropy as a preprocessor. IEEE Trans. Instrum. Meas..

[9] Cannas B, Fanni A, Manetti S, Montisci A, Piccirilli MC (2004). Neural network-based analog fault diagnosis using testability analysis. Neural Comput. Appl..

[10] Hinton GE, Osindero S, Teh YW (2006). A fast learning algorithm for deep belief nets. Neural Comput..

[11] Lee H, Grosse R, Ranganath R, Ng AY (2011). Unsupervised learning of hierarchical representations with convolutional deep belief networks. Commun. ACM..

[12] Chen Z, Li W (2017). Multisensor feature fusion for bearing fault diagnosis using sparse autoencoder and deep belief network. IEEE Trans. Instrum. Meas..

[13] Chen Y, Zhao X, Jia X (2015). Spectral-spatial classification of hyperspectral data based on deep belief network. IEEE J. Sel. Top. Appl. Earth Obs. Remote Sens..

[14] Zhao G, Liu X, Zhang B, Liu Y, Niu G, Hu C (2018). A novel approach for analog circuit fault diagnosis based on deep belief network. Measurement.

[15] Liu Z, Jia Z, Vong C, Bu S, Han J, Tang X (2017). Capturing high-discriminative fault features for electronics-rich analog system via deep learning. IEEE Trans. Ind. Inf..

[16] Xiao Y, He Y (2010). A linear ridgelet network approach for fault diagnosis of analog circuit. Sci. China Inf. Sci..

[17] Cortes C, Vapnik V (1995). Support-vector networks. Mach. Learn..

[18] Cristianini N, Taylor JS (2000). An Introduction to Support Vector Machines and Other Kernel-Based Learning Methods.

[19] Mirjalili S, Mirjalili SM, Lewis A (2014). Greywolf optimizer. Adv. Eng. Softw..

[20] Chen HL, Yang B, Liu J (2011). A support vector machine classifier with rough set-based feature selection for breast cancer diagnosis. Expert Syst. Appl..

[21] Yang C, Tian S, Long B, Chen F (2011). Methods of handling the tolerance and test-point selection problem for analog-circuit fault diagnosis. IEEE Trans. Instrum. Meas..

[22] Shao H, Jiang H, Zhang X, Niu M (2015). Rolling bearing fault diagnosis using an optimization deep belief network. Meas. Sci. Technol..

[23] Chen P, Yuan L, He Y, Luo S (2016). An improved SVM classifier based on double chains quantum genetic algorithm and its application in analogue circuit diagnosis. Neurocomputing.

[24] Yuan X, Miao Z, Liu Z, Yan Z, Zhou F (2020). Multi-strategy ensemble whale optimization algorithm and its application to analog circuits intelligent fault diagnosis. Appl. Sci..

[25] He W, He Y, Li B (2020). Generative adversarial networks with comprehensive wavelet feature for fault diagnosis of analog circuits. IEEE Trans. Instrum. Meas..

